# Report of Committee on Sanitary Science and Police of Pennsylvania State Medical Association

**Published:** 1892-12

**Authors:** Simon J. J. Harger, Francis Bridge, Chalkley H. Magill, J. C. McNeil, J. R. Hart, Wm. B. Werntz, J. H. Timberman

**Affiliations:** University of Pa., Vet. Dep’t, Chairman; Philadelphia, Pa.; Philadelphia, Pa.; Philadelphia, Pa.; Philadelphia, Pa.; Philadelphia, Pa.; Philadelphia, Pa.; Philadelphia, Pa.


### REPORT OF THE COMMITTEE ON SANITARY SCIENCE AND POLICE TO THE PENNSYLVANIA STATE VETERINARY MEDICAL ASSOCIATION.

The task of preparing an exhaustive report on Sanitary and Police is an arduous one; one which, in the face of many disadvantages, deserves by no means to be underrated. Since our last annual meeting in Philadelphia, nothing of significance relative to the subject which has been entrusted to this committee has occurred, or, at least, has not been reported to us. As chairman of this body, I have generously consulted my colaborators, and solicited their suggestions and ideas in the embodiment of this communication. The source of our material has been scattered and somewhat chaotic, and, instead of relating detailed facts and incidents we have confined ourselves rather to generalities. In fact the work of this committee should serve a nobler purpose and have a more useful destiny at this time than the mere enumeration of the number of cases of Texas fever, or rabies, and the discussion of the relation between the bacillus of hog cholera and that of swine plague. Politically speaking, it should be the organ, the voice of this body of veterinarians in executing the good work which lies before us and which the title of the committee signifies. The thorough discharge of its duties involves great responsibility, because of the pecuniary interest to the stock-owner, and the incomparably graver question—that of the health of every individual.

We have arrived at a critical period in sanitary medicine, a period in which the professional man, as well as the layman, recognizes the inefficiency of our sanitary laws and the prevention against disease from one animal to another, and from animal to the human family. The time has come when we may be able to remedy these defects and span over the deep chasm; when we may have some laws passed which will give us greater protection against the fatal ravages of filth and disease.

In securing this result the veterinarian must play no mean part. He, more than anyone else, appreciates the gravity of the situation, from the fact of his comparative pathology and hygiene. It is he who should give impetus to this important work; who should be in the front rank of the scene of fire; and who should be the power behind the throne.

We already have the assistance of our sister profession and of a large number of government and municipal officials. Every one knows the power of the public press as a public educator and as a means of reaching the lesser lights, the multitude. We must enlist the services of our daily newspapers, and encourage articles and editorials to turn the tide of public sympathy in the direction of our cause, and let our principal aim be the benefaction of the public rather than the amelioration of our own professional ambition.

To show that we are not alone in this undertaking, I may state that the Hon. Abraham Beitler, Director of Public Safety of Philadelphia, at present contemplates introducing a bill before the coming State legislature to regulate the inspection of meat in this State. He also anticipates the appointment of a veterinarian on the Board of Health in the near future. Here, then, is the beginning of a line of action by a prominent official, with which our profession should act in unison. The dangers of consuming food-products obtained from diseased animals have been so often discussed and are quite well-known, that it is not necessary to reiterate them here.

The often-asked question as to the laws and remuneration which exist in relation to contagious diseases can be easily summarized. The law of May 9th, 1889, which is the only one governing contagious diseases, is of a general character, and gives the Secretary of the Board of Agriculture the power to disinfect stables and their surroundings, to quarantine herds, and to destroy the animals affected. But the amount appropriated by the commonwealth as a remuneration for such diseased animals as are destroyed after appraisement by sworn appraisers, is the insignificant sum of twenty-five hundred dollars. Think of this amount as a compensation for all the cases of tuberculosis, glanders, and other contagious diseases that exist in the commonwealth of the Keystone State. Since the eradication of contagious pleuro-pneumonia, for which the State officials deserve great credit, about the only disease for which the subject has been officially destroyed is glanders. For such cases the government allows twenty dollars a head. Should the authorities commence a raid on tuberculosis, the indemnity would be practically nothing. True it is, as it is sometimes argued by the more lukewarm sanitarians, that this represents the actual value of the animal in his present condition. Actually he may not be worth one dollar, but we must look a step further. All persons are not equally scrupulous and conscientious. Many are inclined to keep such animals as long as their products will pay for their keep. Some will then sell them or kill them for beef, knowing that they are actually diseased, and have a kinder consideration for their purse than for the public health; others commit the same offense, but it is with ignorance. However, the average farmer and cattle owner is not too self-sacrificing ; he requires an inducement to urge him to report voluntarily the extent of disease among his live stock.

Moreover, as we have good evidence of the contagiousness of the most fatal diseases from animal to man, as this work is intended for the public good and the well-being of the individual, and as it equally concerns each and every one of us.—and what is more precious to us than good health—each State should be willing to make a generous appropriation to give a reasonable remuneration for each animal sacrificed for a public benefit. Economy is wealth, but when practiced in the wrong place is bad management. What is the loss from death of one of the brute creation compared with that of a human being? Yet these two factors often stand in the relation of cause and effect.

In this official capacity, this committee has taken the privilege of addressing to you certain recommendations and suggestions on a few measures which, in their consideration, are very important as well as opportune at the present time. The first one of these subjects is *dairy inspection.* In this step we will undoubtedly meet the greatest opposition and must overcome the greatest obstacles; not altogether from a non-partisan judgment of the case, but also from a certain amount of prejudice which is always attached to similar new and radical measures. Committees to examine this project have been appointed by the Philadelphia Society of Veterinary Medicine and the Philadelphia County Medical Society, but nothing definite has evidently been accomplished. We have in some cities a system of milk inspection whose principal aim seems to be the destruction of water—certainly the least harmful agent with which milk can be adulterated. The tubercle bacillus being found at times in the milk, and even in the butter, and not differing in identity with that of human tuberculosis, and also having sufficient evidence to lead us to believe that tuberculosis is contagious from cow to man, it is clear that the only method to avoid this danger is to go to the source or the fountain-head of the active agent—the dairy. Removing the cause is the only safe procedure. Here again we must avoid that ever-present demon of public appointments—politics.

This committee would suggest a procedure similar in its outlines to the following: Each State may be apportioned into districts, thus limiting the territory of the inspecting veterinarian to a circumscribed area. Each dairy herd should be examined at least twice each year. In the doubtful cases tuberculine may be used, for we see no good reason against its use for this purpose. All animals, discovered to suffer from any diseased condition capable of contaminating the milk so as to render it unfit for consumption, should be destroyed and the stable disinfected. On the the other hand, the inspector should command a respectable salary, and the farmer should receive a reasonable sum for his property which has been confiscated. As regards the appointment of the district veterinarian, it should be by competitive examination, given by a Board of Examiners of three veterinarians appointed by the Governor of the State or the Secretary of the Board of Agriculture. The members of this Board should be reputable and well known in their profession. The examination should be both theoretical as well as practical in the cow stable, because the embryo veterinarian just leaving college, who perhaps never was in a dairy stable, may enumerate the rales and other physical signs more fluently than the experienced practitioner.

Still further, this inspection should not be confined alone to the animal itself, but also to its surroundings and to the dairy utensils. The stable should be maintained in good sanitary condition; the udder should not be a seat of filth; wells and springs should be inspected. Epidemics of typhoid and scarlet fevers and other diseases have been traced to the milk from dairies whose cows received impure water from faulty drainage. Another point which here suggests itself is the production of small-pox virus on cattle that are not thoroughly examined. The inefficiency of private dairy inspection, as practiced by some of our large milk dealers, becomes very evident if we consult their records. Through the kindness of one of my colleagues, I have obtained a statement showing that of two hundred and sixty cows examined only one was found to be suspicious; the others were in good condition.

It should be made obligatory on the part of every stock-owner to report all cases of contagious disease on his premises.

A word as to meat inspection, in which a little more progress has been made. In Philadelphia there are two regular veterinarians appointed as meat inspectors and three detectives, or "spotters," as they are called. Cattle suffering from actinomycosis, tuberculosis and other diseased conditions, were formerly slaughtered without discrimination. Under the present management the carcasses are examined and those unfit for consumption are thrown into the "rendering tank." The same vigilance is pursued in regard to meat offered on the market stall, and a number of successful prosecutions have been made. Although this system has accomplished much, it is evidently defective, and is intended only as a stepping-stone to a higher plane where we will realize our ideal method of inspection. It is simply impossible for two inspectors to visit all the numerous small slaughter houses of a large city, scattered over a territory of miles. The "bologna" butcher is determined to smuggle in his subject if it is possible. What we must have—and the sooner we have it the better—is the abolition of the small slaughter houses, and the establishment of a central abattoir, such as they have in European cities, controlled by the municipality, where all slaughtering must be done. Here the inspecting veterinarian can have supreme control and is able to examine each carcass as the animal is killed. To this abattoir should be attached a laboratory with the necessary outfit to make a microscopic examination of the tissues.

The following official report shows the percentage of diseased carcasses from a given number of animals slaughtered at the Philadelphia abattoir :

FROM JULY 1st, '92, TO JULY 31st, '92.

| | ANIMALS INSPECTED. | | | POST-MORTEMS. | | | | | | | | |
|---|---|---|---|---|---|---|---|---|---|---|---|---|
| | For Export to Great Britain. | For Local Dealers and Butchers. | Southern Cattle for immediate slaughter. | Post-Mortems. | Diseased. | Tuberculo's | Actinomycosis | Non-Contagious Pn'a | Abscess. | Bronchitis. | Bronchial Pneumonia. | Cirrhosis of Liver. |
| 1892 | | | | | | | | | | | | |
| Jan'y . | 2,629 | 17,971 | | 9,820 | 22 | 11 | 4 | 4 | 2 | | | 1 |
| Feb'y. | 3,003 | 13,477 | | 8,392 | 31 | 22 | 4 | 2 | 3 | | | |
| March. | 3,004 | 12,910 | | 9,444 | 35 | 32 | | 1 | 1 | | | 1 |
| April.. | 3,496 | 14,551 | | 8,900 | 23 | 18 | 2 | 2 | 1 | | | |
| May .. | 3,373 | 14,824 | 44 | 9,959 | 17 | 6 | 9 | 1 | | 1 | | |
| June .. | 3,910 | 12,365 | 1,998 | 9,199 | 13 | 8 | 4 | | | | 1 | |
| July... | 3,463 | 11,716 | 4,451 | 8,251 | 0 | | | | | | | |
| | | | | 63,965 | 141 | 97 | 23 | 10 | 7 | 1 | 1 | 2 |

The necessity of a closer inspection of our food-stuffs in general is becoming more apparent, and it will be only a question of time and perseverence when we will receive the proper protection in this direction. One of the members of this committee has made the important suggestion that a State Bacteriological Laboratory, for the investigation of diseases under a competent director, be established at the Veterinary Department of the University of Pennsylvania, and that a generous appropriation be made for the same. Of course we cannot expect to obtain everything in one moment, but if each one play well the part which belongs to him, we will advance steadily and finally reach the coveted goal.

It is gratifying to say that there has been no serious outbreak of contagious or infectious diseases among the domestic animals of this State during the past year. Not a single case of contagious pleuro-pneumonia has been found. Many supposed cases of this disease have been reported, but, upon investigation, have been found to be some other trouble. The greatest possible vigilance has been exercised by the Bureau of Animal Industry and the State Board of Agriculture to prevent the introduction of any contagious or infectious disease from other States or countries.

Tuberculosis among dairy stock has been the most troublesome contagious disease with which we had to deal. A large number of cases have been reported to the State authorities, but no action has been taken by the State, further than to advise the destruction of affected animals; in such cases in which this advice was not followed, quarantining the affected animals was adopted, the small appropriation by the State for the destruction of animals affected with contagious diseases being too small to pay for the large number of animals affected with this disease.

The number of cattle affected has been principally among the Jerseys.

*Texas Fever*—Since the regulation of the transportation of cattle from the Gulf States by the United States authorities, few cases of Texas fever have been reported; there were but eleven outbreaks during the past year.

*Actinomycosis*—A number of animals affected with this disease have been destroyed, their carcasses being considered unfit for food, and the disease itself contagious. While most authorities are inclined to think if the animal is otherwise healthy that the removal of the head is sufficient protection against any injurious effects ; this conclusion is, nevertheless, not by all considered solved.

*Glanders*—Eighty-four cases of glanders have been reported during the last year, but this does not represent all the cases which have existed. Many of these cases, after careful examination and inoculation of guinea pigs with the nasal discharge, have been discovered not to be glanders. When the cases have been well marked, or the inoculations have proved the existence of the disease, the animals have been destroyed and their owners compensated by sums varying from five to twenty-five dollars, according to condition and surroundings. This compensation is not sufficient to induce unscrupulous persons, who may unfortunately own affected animals, to report this fact to a veterinarian, but will rather try to dispose of them to better advantage and thus spread the disease. It would be better economy to pay a fair price and thus remove the temptation to this disgraceful practice.

*Influenza*—There have been fewer cases of influenza among horses in the State than for several years, but where it has existed there was a greater tendency to pleurisy and pneumonia.

*Anthrax*—Fewer cases of this disease have been reported in this State than for several years past, probably owing to the exceptionally dry weather which is not favorable to the development of anthracoid diseases. An outbreak in several herds has been very recently reported, and, in fact, still exists in Delaware, and if treated without care may spread into our own State.

Simon J. J. Harger, University of Pa., Vet. Dep't, *Chairman.*

Francis Bridge.

Chalkley H. Magill.

J. C. McNeil.

J. R. Hart.

Wm. B. Werntz.

J. H. Timberman.

Philadelphia, Pa., Sept. 5, 1892.

